# Improving Compliance With Royal College of Surgeons Handover Standards: A Prospective Clinical Audit and Quality Improvement Project

**DOI:** 10.7759/cureus.117993

**Published:** 2026-10-15

**Authors:** Omar Nasir, Florencia Vecchio

**Affiliations:** 1 Urology, Colchester General Hospital, East Suffolk and North Essex NHS Foundation Trust, Colchester, GBR

**Keywords:** clinical audit, clinical handover, closed-loop audit, patient safety, quality improvement, surgical education, urology

## Abstract

Background: Effective handover of clinical information is essential to patient safety, particularly in surgical specialties where care is delivered across shift patterns. We undertook a prospective, closed-loop clinical audit and quality improvement project with the primary objective of determining whether a structured handover intervention improved compliance with Royal College of Surgeons (RCS) of England minimum handover standards. Secondary objectives were to characterise handover practices and information-transfer vulnerabilities across daytime intra-specialty urology handover and overnight inter-specialty cross-cover to general surgery, and to evaluate the uptake and impact of the resulting intervention.

Methodology: Handover practice in a single urology department (Colchester Hospital, East Suffolk and North Essex NHS Foundation Trust) was audited against six RCS of England minimum data criteria (patient name and age, date of admission, ward location, responsible consultant, current diagnosis, and results of significant or pending investigations), each scored out of six. Cycle 1 evaluated 12 handovers between March 15 and April 15, 2025. As urology inpatients are covered overnight by general surgery on-call Foundation Year 1 (FY1) doctors rather than urology-specific junior staff, a cross-sectional survey of 10 FY1s was undertaken, alongside assessment of the timing of overnight-to-day registrar handover. Findings were presented at the local audit meeting and informed a quality improvement intervention comprising an updated written handover template, a colour-coded patient stratification tool, a 07:30 deadline for flagging new patients prior to the daytime handover, and induction teaching for incoming FY1 doctors. Cycle 2 (re-audit) repeated the same assessment over 12 handovers (July 10-August 10, 2025); a combined cohort of 280 patient handovers was assessed across both cycles. Daily mean scores were compared between cycles using a paired-samples t-test.

Results: The mean handover score improved from 4.39/6 (73.2%) in cycle 1 to 5.37/6 (89.5%) in cycle 2, a statistically significant increase (mean difference 0.98 points, 95% CI 0.64-1.31; paired t(11) = 6.38, p < 0.0001; Cohen’s d = 1.84). The greatest gains were in documentation of the responsible consultant surgeon (49.2% (58/118) to 79.2% (133/168)) and communication of significant or pending investigation results (71.2% (84/118) to 95.8% (161/168)); 75 pending investigations, predominantly imaging, had been undocumented at cycle 1. Night registrar-to-day handover time improved from a mean of 07:37 to 07:15. Despite introduction of a colour-coded handover stratification tool, day-to-night handover remained purely verbal with no written record in either cycle, and on-call FY1s continued to report feeling inadequately prepared to assess deteriorating urology patients overnight.

Conclusions: A standardised written handover proforma and an earlier registrar handover time produced a statistically significant, clinically meaningful improvement in compliance with RCS of England handover standards in a busy urology department. Day-to-night handover to on-call foundation doctors remained unstructured in practice despite a dedicated stratification tool, suggesting possible implementation barriers rather than a flaw in intervention design, although the specific reasons for non-adoption were not formally investigated; a third audit cycle assessing uptake of this tool is recommended.

## Introduction

Handover of clinical information between shifts is a recognised point of vulnerability in patient safety: unstructured or incomplete handovers are associated with a higher risk of adverse events, near misses, and critical omissions in ongoing care planning [1,2]. In UK surgical practice, this risk was heightened by the introduction of the European Working Time Directive, which increased shift-based working and, consequently, the frequency of handovers between clinical teams [3]. In response, the Royal College of Surgeons (RCS) of England published minimum standards for safe handover, requiring that a patient’s name and age, date of admission, ward location, responsible consultant, current diagnosis, and results of any significant or pending investigations are reliably communicated at every handover [3].

Structured, proforma-based handover tools have been shown to improve compliance with minimum handover data sets in other surgical populations [4], and national guidance recommends structured handover as part of routine acute and emergency surgical care, citing reductions in critical data omissions and improved staff satisfaction where it is properly implemented [5]. Maintaining continuity of care through effective handover is also an explicit professional duty under Good Medical Practice (GMC) [6]. Despite this evidence base, handover practice remains highly variable in the surgical literature, and is infrequently subject to iterative, closed-loop quality improvement within individual surgical subspecialties such as urology [2].

Within our institution, these risks are amplified by the local staffing and escalation model. During normal working hours, urology inpatients are managed by a dedicated team comprising a Foundation Year 1 (FY1) doctor, senior house officer (SHO), specialist registrar (SpR), and consultant. At the end of the day, responsibility for outstanding clinical tasks is transferred from the urology FY1 to the general surgery on-call FY1, who provides overnight ward cover for urology patients. Although the urology SpR remains resident overnight, they become involved primarily through referral rather than routine patient review. Consequently, the general surgery on-call FY1 serves as the initial responder for deteriorating urology inpatients. As this doctor is often unfamiliar with urology-specific conditions, management plans, and ongoing investigations, a comprehensive and accurate handover represents the principal safeguard for maintaining continuity of care and ensuring timely, appropriate clinical decision-making.

We undertook a prospective, closed-loop clinical audit and quality improvement project (QIP) in a urology department. The primary objective was to determine whether implementation of a structured handover intervention improved compliance with RCS of England minimum handover standards, thereby improving the quality and safety of information transfer between clinical teams. Secondary objectives were to (i) characterise handover practices and identify information-transfer vulnerabilities, distinguishing between daytime intra-specialty urology handover and overnight inter-specialty cross-cover to general surgery on-call FY1 doctors, and (ii) implement and evaluate the uptake and impact of a targeted handover intervention package.

## Materials and methods

This project was designed, conducted, and is reported in accordance with the SQUIRE 2.0 (Standards for Quality Improvement Reporting Excellence) guidelines [7]. This was a prospective, closed-loop clinical audit and QIP undertaken at Colchester Hospital’s Urology Department, East Suffolk and North Essex NHS Foundation Trust, and registered with the trust’s clinical audit department (registration number SGA24-114). Handover practice was measured against the minimum standards for safe handover published by RCS of England [3], comprising six mandatory data items: patient name and age, date of admission, ward and bed location, the responsible consultant surgeon, current diagnosis, and the results of any significant or pending investigations. Each patient handover was scored out of six according to the number of criteria documented and/or verbally conveyed; a more detailed breakdown additionally recorded compliance with each individual identifier, including date of birth, as a discrete data point. A single audited handover was defined at the level of the individual patient: every patient on the daily handover list was scored individually against the six criteria, giving 118 patient-level handovers in cycle 1 and 168 in cycle 2, with the daily score for each audit day calculated as the mean of the per-patient scores for all patients handed over that day. Scoring of each criterion was binary (present/absent) rather than partial: a criterion was scored as present if it was conveyed through either the written list or verbally, individually or in combination, and discordance between the written and verbal account of the same criterion was not separately recorded.

Within the department, urology inpatients are covered by the general surgery on-call FY1 overnight, who is the first point of clinical escalation for any unwell or deteriorating patient; the on-site urology SpR is involved on a referral basis rather than through routine review. Accurate, complete handover of urology-specific information to this FY1 is therefore particularly important to safe overnight care.

Cycle 1: baseline audit

Data were collected prospectively over 12 handovers between March 15, 2025, and April 15, 2025. Data collection was undertaken by more than one member of the audit team, who cross-checked entries informally; a formal inter-rater reliability statistic was not calculated. Handover content was assessed from the written handover list (maintained on a shared drive) together with information conveyed verbally, captured directly by an auditor present at the time of handover, cross-referenced against the electronic patient record (EPR) systems in use at the trust (WebICE, Sentinel, and CareFlow). In parallel, a cross-sectional survey of 10 general surgery on-call FY1s assessed the format and subjective adequacy of the day-to-night and night-to-day handover of urology patients using an anonymous, structured four-item questionnaire asking (1) whether a handover was received (yes/no); (2) the form of handover received (verbal/written/both); (3) whether the respondent had been called to assess an unwell urology patient overnight (yes/no); and, if so, (4) how well-informed they felt in handing over that patient, rated on a five-point scale (5 = well informed). A fresh sample of 10 FY1s, independent of the cycle 1 sample, was surveyed in cycle 2, and the time at which the night SpR handed over to the day team was recorded for each audit day.

Intervention

Findings from cycle 1 were presented at the department’s local audit meeting. Following this, a QIP comprising four linked changes was implemented by the audit team in conjunction with the supervising consultant urologists, targeting the criteria and handover pathways identified as weakest.

First, an updated written handover template - the “Urology List” (Figure 1) - was introduced on the shared drive, built around the RCS of England minimum data set; a fixed reminder banner specified that every entry must record (i) patient name and date of birth, (ii) date of admission, (iii) location, (iv) responsible consultant, (v) diagnosis, and (vi) past medical history, results, antibiotics, and pending investigations. Second, a colour-coded (“traffic light”) system (Figure 2) was introduced to stratify the acuity of patients being handed over from the day team to the night on-call team, for use by both FY1s and registrars (SpRs): patients were marked green if newly admitted and clinically stable, amber if listed for theatre - with the associated pre-operative checklist (X-ray ordered, consent obtained, and operative site marked) tracked within the same colour band - and red if clinically unwell and requiring closer overnight monitoring. Third, the on-call SpR was required to inform the team of all new patients to be added to the handover list by 07:30, giving the FY1 enough time to gather the correct clinical information and update the handover list accurately ahead of the 08:00 day handover. Fourth, incoming general surgery and urology FY1s were to be taught the importance of accurate handover lists, escalation pathways, and troubleshooting, at induction, led by SHOs.

**Figure 1:**
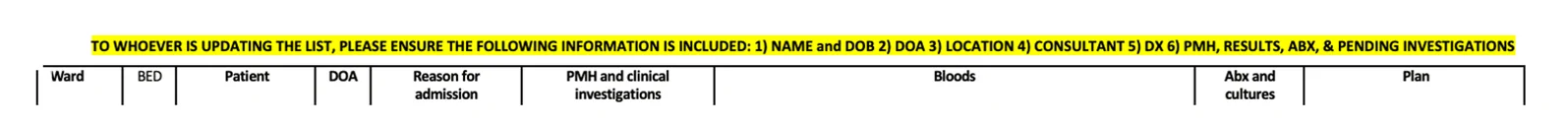
Reminder banner from the standardised “Urology List” handover proforma introduced between audit cycles, specifying the mandatory data fields to be completed for every patient.

**Figure 2:**
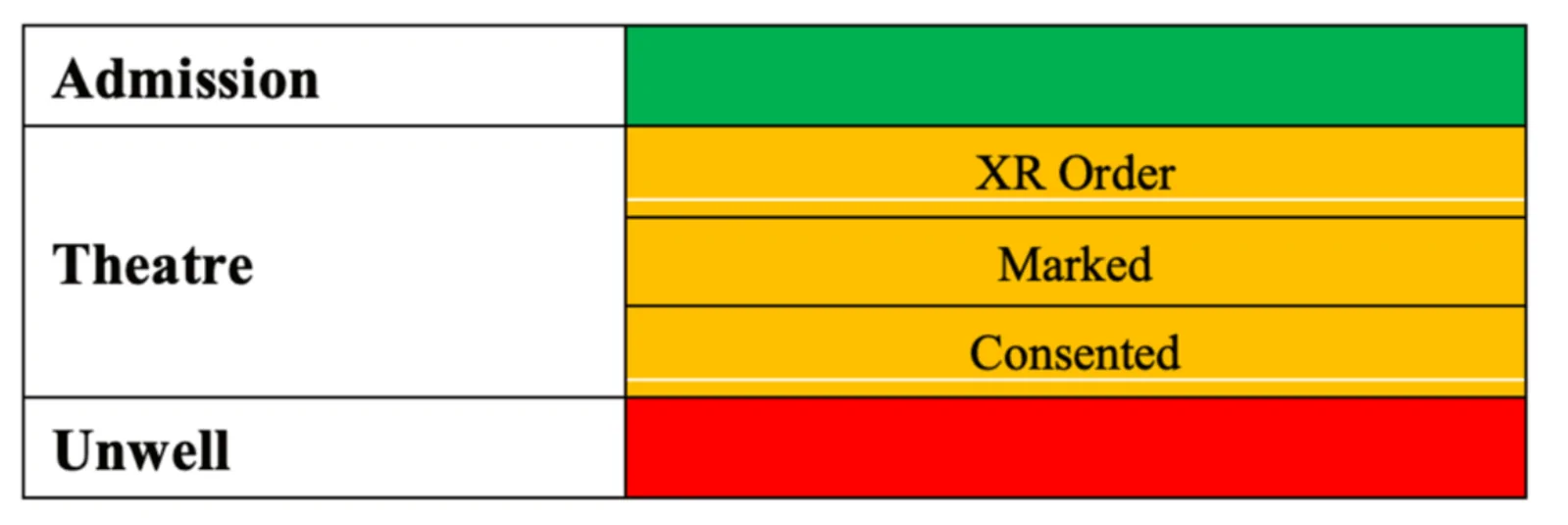
Colour-coded traffic light key applied to the handover list to stratify patient status: green = newly admitted and stable; amber = listed for theatre, with the pre-operative checklist (X-ray ordered, consent obtained, and site marked) tracked in the same band; red = clinically unwell and requiring closer overnight monitoring.

Cycle 2: re-audit

The same assessment was repeated over 12 consecutive handovers between July 10, 2025, and August 10, 2025, using an identical scoring method and data sources. The FY1 survey and night registrar handover timing were reassessed using the methodology described for cycle 1. Across both cycles, a combined cohort of 280 individual patient handovers was assessed.

Outcome measures

The primary outcome was the mean per-patient handover score (out of six) for each audit day, and the percentage compliance with each individual RCS of England criterion. Secondary outcomes were the number and nature of undocumented significant or pending investigations, the timing of night registrar-to-day handover, and FY1-reported adequacy of overnight urology handover.

Statistical analysis

Descriptive statistics (mean, percentage) were calculated for each audit cycle and for each individual criterion. The 12 daily mean handover scores from cycle 1 were compared with the 12 daily mean handover scores from cycle 2 using a two-tailed paired-samples t-test (scipy.stats.ttest_rel), with statistical significance set at p < 0.05. Effect size was reported as Cohen’s d. Analyses were performed in Python 3.13 (SciPy 1.18; NumPy 2.5; Python Software Foundation, Fredericksburg, VA, USA). All 12 paired daily observations were complete in both cycles, with no missing data. Normality of the paired differences was assessed using the Shapiro-Wilk test (W = 0.90, p = 0.17), supporting the use of a parametric paired t-test.

## Results

Cycle 1: baseline findings

Across the 12 audit days in cycle 1, the mean handover score was 4.39/6 (73.2%), ranging from 3.45/6 (58%) to 5.00/6 (83%) (Table 1; Figure 3). Compliance was lowest for documentation of the responsible consultant surgeon (49.2%, 58/118) and patient name and age (42.4%, 50/118), and highest for patient name and date of birth and current diagnosis (both 95.8%, 113/118) (Table 2; Figure 4). Seventy-five significant or pending investigations were not documented at handover, the majority relating to pending imaging. The FY1 survey (n = 10) found that day-to-night handover of urology patients was predominantly verbal with no formal written record; the majority of FY1s reported receiving no handover of, or feeling inadequately informed about, unwell urology patients. The mean time of night registrar handover to the day team was 07:37 (Table 3).

**Table 1:** Mean patient handover score (out of six) and percentage compliance, by audit day, cycle 1 versus cycle 2.

Audit day	Cycle 1 mean score (/6)	Cycle 1 (%)	Cycle 2 mean score (/6)	Cycle 2 (%)
1	3.73	62.2%	5.35	89.2%
2	4.72	78.7%	5.56	92.7%
3	4.90	81.7%	5.64	94.0%
4	3.45	57.5%	5.41	90.2%
5	4.44	74.0%	5.05	84.2%
6	3.78	63.0%	5.47	91.2%
7	4.43	73.8%	5.63	93.8%
8	4.52	75.3%	5.56	92.7%
9	4.30	71.7%	4.94	82.3%
10	5.00	83.3%	5.35	89.2%
11	4.71	78.5%	5.28	88.0%
12	4.75	79.2%	5.21	86.8%
Mean	4.39	73.2%	5.37	89.5%

**Figure 3:**
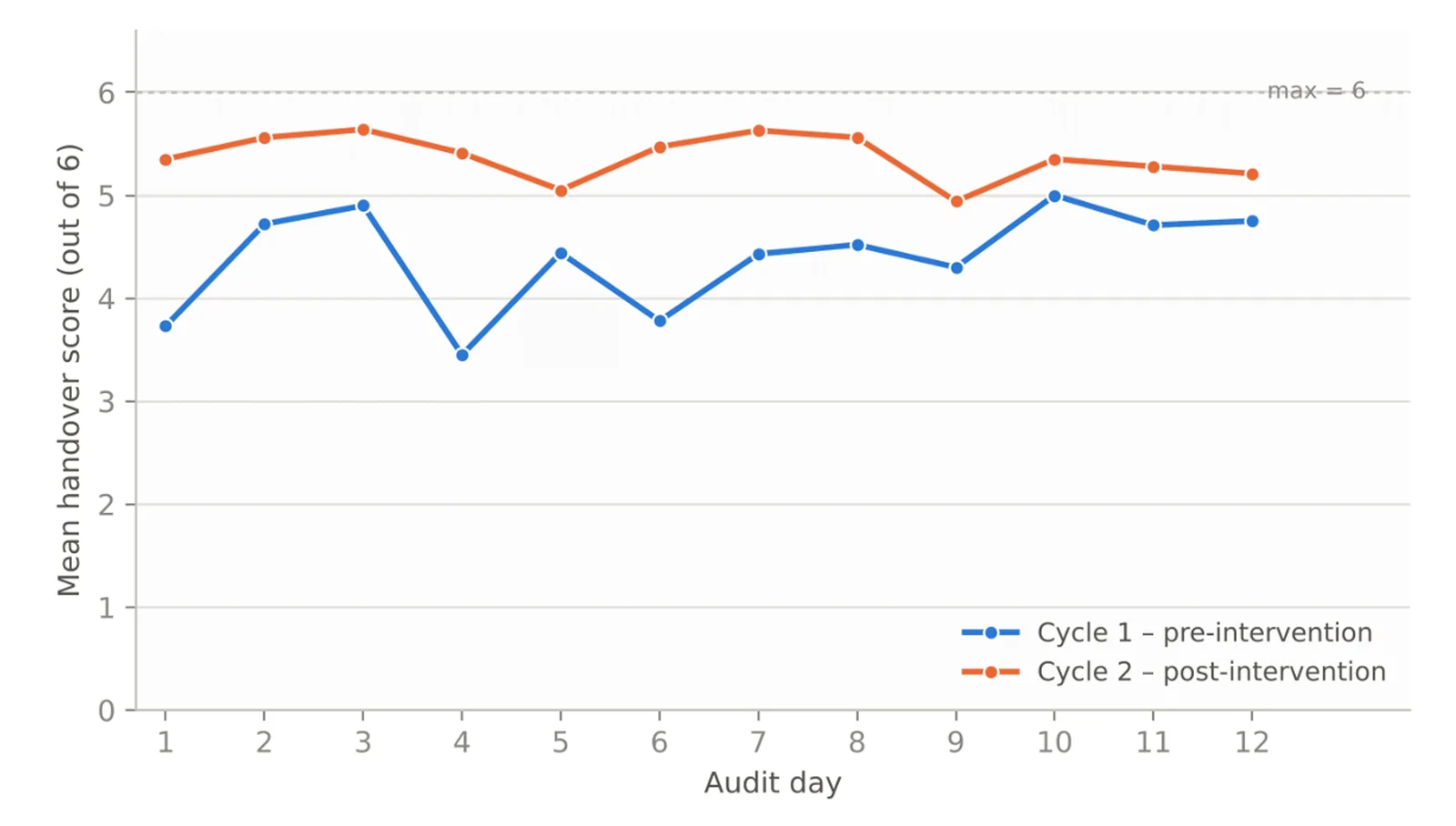
Daily mean handover score (out of six) across the 12 audit days of cycle 1 (pre-intervention) and cycle 2 (post-intervention).

**Table 2:** Count and percentage compliance with individual RCS of England handover criteria, cycle 1 (n = 118 patients) versus cycle 2 (n = 168 patients). RCS: Royal College of Surgeons

RCS of England criterion	Cycle 1 (%)	Cycle 2 (%)
Patient name and age	50 (42.4%)	150 (89.3%)
Patient name and date of birth	113 (95.8%)	153 (91.1%)
Date of admission	105 (89.0%)	149 (88.7%)
Location (ward and bed)	111 (94.1%)	158 (94.0%)
Responsible consultant surgeon	58 (49.2%)	133 (79.2%)
Current diagnosis	113 (95.8%)	156 (92.9%)
Results of significant or pending investigations	84 (71.2%)	161 (95.8%)

**Figure 4:**
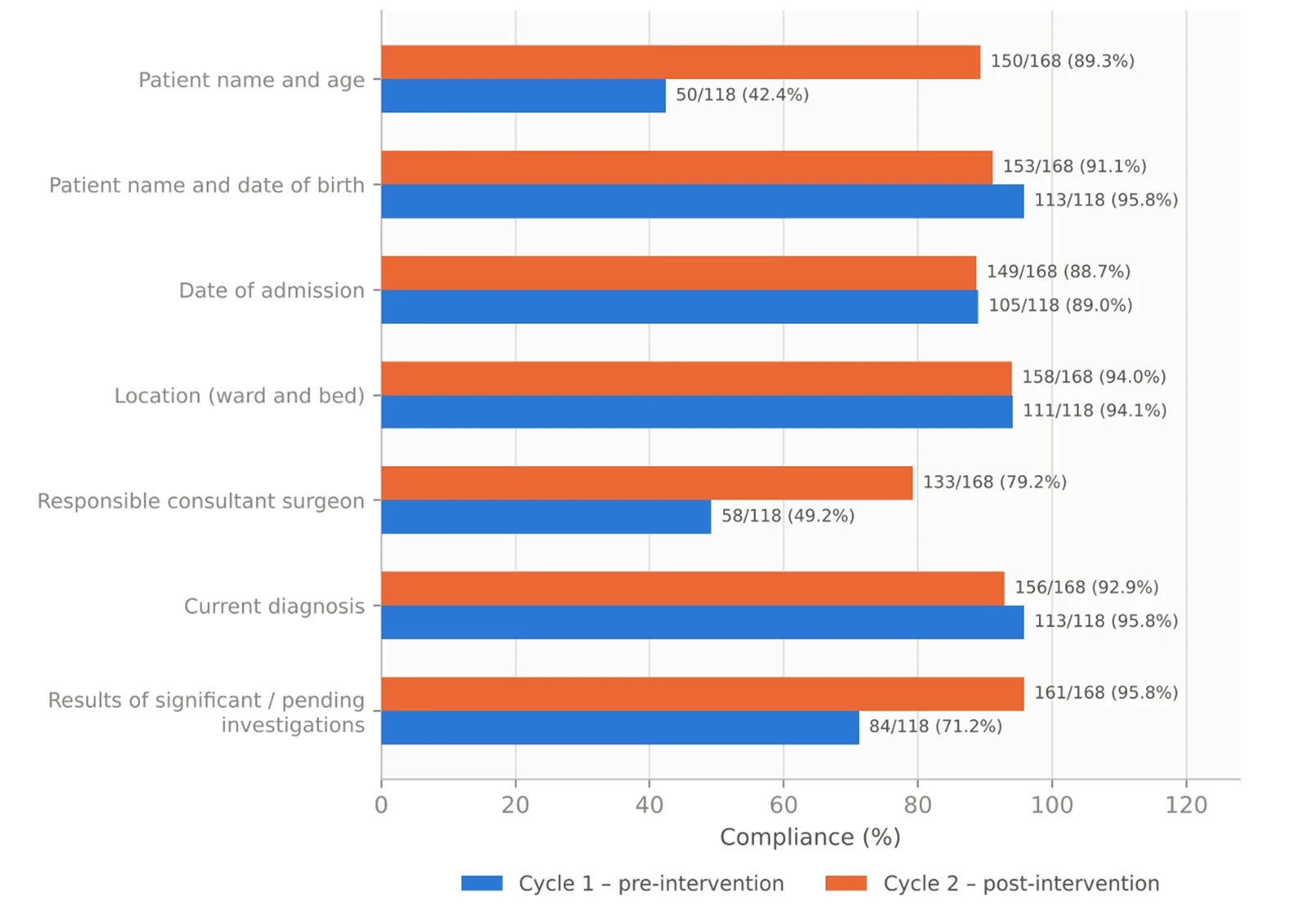
Compliance (count and percentage) with individual RCS of England handover criteria, cycle 1 (pre-intervention, n = 118) versus cycle 2 (post-intervention, n = 168). RCS: Royal College of Surgeons

**Table 3:** Day-to-night FY1 handover and night registrar (SpR) handover timing, cycle 1 versus cycle 2. FY1: Foundation Year 1; SpR: specialist registrar

Metric	Cycle 1 (pre-intervention)	Cycle 2 (post-intervention)
Mean night SpR-to-day-team handover time	07:37	07:15
Format of day-to-night FY1 handover	Predominantly verbal; no formal written record	Verbal only; no written record introduced
FY1-reported adequacy assessing unwell urology patients overnight	Majority reported no handover received, or felt inadequately informed	Majority continued to report feeling uninformed and unprepared
Survey sample	10 general surgery on-call FY1s	10 general surgery on-call FY1s

Cycle 2: re-audit findings (post-intervention)

Following introduction of the updated handover template and associated QIP actions, the mean handover score improved to 5.37/6 (89.5%), ranging from 4.94/6 (82%) to 5.64/6 (94%) (Table 1; Figure 3). Compliance improved across six of the seven individual criteria assessed (Table 2; Figure 4), most markedly for documentation of the responsible consultant surgeon (49.2% (58/118) to 79.2% (133/168)) and results of significant or pending investigations (71.2% (84/118) to 95.8% (161/168)). Documentation of date of admission was virtually unchanged (89.0% (105/118) to 88.7% (149/168)), and recording of patient name and date of birth fell marginally (95.8% (113/118) to 91.1% (153/168)). The mean night registrar-to-day handover time improved to 07:15, consistent with the new 07:30 deadline for flagging new patients ahead of the 08:00 day handover. However, the FY1 survey (n = 10) found that day-to-night handover of urology patients remained exclusively verbal, with no written or colour-coded record evident in practice; despite the introduction of the traffic light stratification tool for this pathway, general surgery FY1s continued to report feeling uninformed about urology patients and unprepared to assess deteriorating patients overnight (Table 3).

Statistical comparison

The improvement in mean daily handover score between cycle 1 and cycle 2 was statistically significant (mean difference 0.98 points, 95% CI 0.64-1.31; paired t(11) = 6.38, p < 0.0001), with a large effect size (Cohen’s d = 1.84).

## Discussion

This closed-loop audit demonstrates that a simple, low-cost intervention - a standardised written handover proforma combined with an earlier registrar handover time intended to give the FY1 preparing the list enough time to gather accurate clinical information before the 08:00 handover - produced a statistically significant and clinically meaningful improvement in compliance with RCS of England minimum handover standards in a urology department, consistent with existing evidence that structured, proforma-based handover tools improve documentation compliance in other surgical populations [4], and that structured handover more broadly is associated with fewer critical data omissions [5].

The largest gains were seen in documentation of the responsible consultant surgeon and communication of significant or pending investigation results - arguably the two criteria of greatest immediate patient safety relevance, since ambiguity over consultant responsibility can delay escalation, and 75 undocumented pending investigations (predominantly imaging) in cycle 1 represented a tangible risk of delayed diagnosis or follow-up. The fact that both criteria improved substantially (49.2% (58/118) to 79.2% (133/168), and 71.2% (84/118) to 95.8% (161/168), respectively) suggests the proforma’s explicit prompts were effective in targeting previously under-recorded fields, consistent with the taxonomy proposed by Ryan et al., in which structured documentation tools were among the more consistently effective handover interventions identified across surgical specialties [2].

These findings are consistent with other UK surgical QIPs that introduced a structured handover proforma to target comparably low baseline compliance. Din et al., at Ashford and St Peter’s Hospitals NHS Foundation Trust, implemented a written weekend surgical handover tool incorporating an identical colour-coded (“traffic light”) acuity stratification system to the one introduced in this project, and reported an increase in the proportion of patients with a completed handover proforma from 35% to 85% following its introduction [8]. Similarly, Bradley, auditing a general surgical ward at NHS Tayside against the same RCS of England minimum handover criteria used in the present project, found that introduction of a structured handover tool increased the proportion of patients receiving a formal handover from 15% to 45%, with compliance with the required information content rising from 0% to 100% among those handed over [9]. The magnitude of improvement across these three UK surgical projects - this audit, Din et al., and Bradley - is broadly comparable in the weakest-performing criteria, and their convergence, each introducing a structured written handover tool in a different NHS surgical department, lends support to the conclusion that the improvement observed here reflects a reproducible effect of structured proforma design, rather than a feature specific to this department or patient population.

Beyond its role in escalation, unambiguous documentation of the responsible consultant surgeon has direct implications for the continuity and efficiency of a patient’s wider admission. Where responsibility is not clearly recorded, patients are at greater risk of being reviewed, and their management plans amended, by more than one consultant across a single admission, which can result in duplicated investigations, conflicting management plans, and delayed or fragmented decision-making across the day-night-day handover cycle. A single, clearly identified responsible consultant is also the mechanism by which post-discharge follow-up is organised, ensuring patients are followed up under one consistent clinical lead rather than being lost between teams; this is consistent with GMC guidance that continuity of care is a professional duty [6], and with wider clinical governance guidance that a named clinician or team should formally take over responsibility for a patient before handover is considered complete [10]. The improvement in this criterion from 49.2% (58/118) to 79.2% (133/168) is therefore likely to have benefits extending beyond the immediate handover encounter to the coherence of the patient’s overall admission and discharge pathway; nevertheless, at 79.2% compliance (133/168), around one in five handovers in cycle 2 still lacked this information, indicating that this risk, whilst reduced, was not eliminated.

Compliance with date of admission was virtually unchanged (89.0% (105/118) in cycle 1 vs. 88.7% (149/168) in cycle 2), and recording of date of birth fell marginally (95.8% (113/118) to 91.1% (153/168)); both criteria were already close to ceiling at baseline, and this small residual variation more likely reflects occasional transcription gaps than a failure of the intervention itself.

Despite these improvements, the day-to-night handover of urology inpatients to the general surgery on-call FY1 responsible for overnight cover remained a concern, despite the implementation of a targeted intervention. As the FY1 is not urology-specific, they represent the first point of clinical escalation for any deteriorating urology inpatient, with assessment and initial referral occurring before direct involvement of the on-site urology SpR, who is engaged primarily on a referral basis. Consequently, the overnight FY1 is particularly dependent on the accuracy and completeness of the handover to safely recognise and manage patients with whom they may be unfamiliar. The QIP explicitly addressed this pathway through a colour-coded traffic light tool intended to stratify patient acuity for FY1s and SpRs at the point of day-to-night handover, a 07:30 deadline for flagging new patients ahead of the 08:00 day handover, and dedicated SHO-led induction teaching for incoming FY1s. Nevertheless, the cycle 2 FY1 survey found this handover remained purely verbal, with no written or colour-coded record evident in practice, and FY1s continued to report feeling uninformed and unprepared to assess deteriorating urology patients overnight. This discrepancy between the intended design of the intervention and its observed use is suggestive of possible implementation barriers rather than a flaw in the intervention’s design, although the specific reasons for non-adoption were not formally investigated in this audit; possible explanations include incomplete rollout or awareness of the traffic light tool among the FY1 cohort surveyed in cycle 2, induction teaching not yet having reached doctors already mid-rotation at the time of re-audit, or a persistent cultural preference for verbal-only handover that a documentation tool alone does not overcome. This is consistent with the wider literature: verbal-only handover, even where a structured alternative exists, is recognised as more vulnerable to loss of information than a combined verbal-and-written approach [1,2], and WHO patient safety guidance specifically recommends a structured, written adjunct to verbal handover wherever information is time-critical [1]. It also underlines that structured tools require active reinforcement and audit to achieve real-world adoption, not simply introduction [5]. This represents the most important target for the recommended third audit cycle, which should specifically assess uptake and use of the traffic light tool, not only its existence. Beyond reinforcement and audit alone, a more durable solution may lie in changing the mechanism of documentation itself: a shared-drive document remains an easily bypassed adjunct under time pressure, outside clinicians’ usual workflow and invisible to anyone not actively seeking it out. Embedding the colour-coded stratification as a mandatory, structured field within the hospital’s EPR system - rather than a separate shared-drive document - would integrate it directly into the existing day-to-night handover workflow, make non-completion visible to the wider team in real time, and allow uptake to be tracked automatically rather than relying solely on survey-based assessment. This EPR-based approach is recommended as a specific target for implementation and evaluation in the third audit cycle.

This audit assessed process compliance - whether the required information was documented and communicated - rather than downstream patient-level outcomes. The strongest evidence that structured handover translates into measurable safety benefit, rather than simply more complete documentation, comes from the multicentre I-PASS study, in which implementation of a standardised handoff bundle - comprising a mnemonic handoff framework, structured verbal handoff training, and a written handoff tool - across nine paediatric residency programmes was associated with a 23% relative reduction in medical errors and a 30% relative reduction in preventable adverse events, without increasing handoff duration [11]. Although I-PASS was developed and validated in paediatric inpatient medicine rather than adult general surgery, its scale and design provide the strongest available evidence that a structured, written handover tool of the kind introduced in this project can plausibly be expected to reduce patient harm and not only improve documentation completeness; confirming whether a comparable reduction in adverse events follows from the improved compliance demonstrated here would require a further, outcomes-focused audit cycle linking handover completeness to downstream clinical incidents. The sustainability of these improvements over time was not assessed within this project; continued compliance is likely to depend on embedding the proforma into departmental induction and routine practice, as recommended below.

Limitations

This audit has several limitations. It was conducted at a single centre within a single surgical subspecialty, which may limit generalisability to other units or specialties. Each cycle comprised 12 handover days over a one-month period which, whilst consistent with standard closed-loop audit practice, is a relatively short observation window that may be susceptible to staffing and seasonal variation; the two cycles were also separated by several months, during which factors unrelated to the intervention - most notably rotational turnover of junior medical staff (FY1s and SpRs), who in the UK rotate onto and off placements at fixed points in the year - could have contributed to the observed improvement, independent of the intervention itself. The handover-scoring tool was developed in-house for this project and has not been externally validated, as was the FY1 survey instrument, which has similarly not undergone formal psychometric validation; conclusions regarding subjective FY1 preparedness should therefore be interpreted with more caution than the handover compliance findings, and data collection was not blinded to the audit cycle, introducing a risk of observer bias, particularly for the more subjectively assessed FY1 survey responses (n = 10 in each cycle). Although data were cross-checked informally between the assessors involved, a formal inter-rater reliability statistic was not calculated. Intervention fidelity was not separately tracked: uptake of the written proforma and the traffic light tool was inferred only from the cycle 2 re-audit compliance scores and the FY1 survey’s handover-format question, rather than from direct, real-time monitoring of proforma completion, induction-teaching attendance, or tool use. Per-criterion compliance was reported descriptively; because individual-patient-level counts for each criterion were not retained separately within the audit dataset, formal significance testing was performed only on the day-level mean handover score, not on each individual criterion. This also means the effective sample size for the primary statistical comparison was the 12 paired daily observations per cycle, rather than the 280 individual patient handovers assessed overall, which limits statistical power and increases susceptibility to day-to-day variation. Finally, this audit measured process compliance (documentation and communication of information) rather than patient-level outcomes, and no attempt was made to link handover omissions directly to clinical harm.

## Conclusions

In this prospective, closed-loop clinical audit and QIP, introduction of an updated written handover template, alongside a traffic light stratification tool, a 07:30 deadline for flagging new patients, and induction teaching produced a statistically significant improvement in overall compliance with RCS of England minimum handover standards in a urology department, with the greatest gains in documentation of the responsible consultant and communication of pending investigation results. Day-to-night handover of on-call foundation doctors remained unstructured in practice despite a tool specifically designed for this pathway, and foundation doctors continued to report feeling unprepared to assess deteriorating patients overnight - suggesting possible implementation barriers rather than a flaw in intervention design, although the specific reasons for non-adoption were not formally investigated. We recommend reinforcing awareness and use of the traffic light handover tool through induction teaching already introduced, and undertaking a third audit cycle to confirm sustained improvement and measure uptake of this tool specifically.
